# Misfolded Proteins: From Little Villains to Little Helpers in the Fight Against Cancer

**DOI:** 10.3389/fonc.2015.00047

**Published:** 2015-02-24

**Authors:** Ansgar Brüning, Julia Jückstock

**Affiliations:** ^1^Molecular Biology Laboratory, Ludwig-Maximilians-University, Munich, Germany

**Keywords:** proteasome, autophagy, bortezomib, nelfinavir, endoplasmic reticulum stress, aggresome, HDAC6

## Abstract

The application of cytostatic drugs targeting the high proliferation rates of cancer cells is currently the most commonly used treatment option in cancer chemotherapy. However, severe side effects and resistance mechanisms may occur as a result of such treatment, possibly limiting the therapeutic efficacy of these agents. In recent years, several therapeutic strategies have been developed that aim at targeting not the genomic integrity and replication machinery of cancer cells but instead their protein homeostasis. During malignant transformation, the cancer cell proteome develops vast aberrations in the expression of mutated proteins, oncoproteins, drug- and apoptosis-resistance proteins, etc. A complex network of protein quality-control mechanisms, including chaperoning by heat shock proteins (HSPs), not only is essential for maintaining the extravagant proteomic lifestyle of cancer cells but also represents an ideal cancer-specific target to be tackled. Furthermore, the high rate of protein synthesis and turnover in certain types of cancer cells can be specifically directed by interfering with the proteasomal and autophagosomal protein recycling and degradation machinery, as evidenced by the clinical application of proteasome inhibitors. Since proteins with loss of their native conformation are prone to unspecific aggregations and have proved to be detrimental to normal cellular function, specific induction of misfolded proteins by HSP inhibitors, proteasome inhibitors, hyperthermia, or inducers of endoplasmic reticulum stress represents a new method of cancer cell killing exploitable for therapeutic purposes. This review describes drugs – approved, repurposed, or under investigation – that can be used to accumulate misfolded proteins in cancer cells, and particularly focuses on the molecular aspects that lead to the cytotoxicity of misfolded proteins in cancer cells.

## Introduction: How Do Proteins Fold and What Makes Misfolded Proteins Dangerous?

For an understanding of misfolded proteins, it is necessary to understand how cellular proteins attain and then further maintain their native conformation and how mature proteins and unfolded proteins are generated and converted into each other.

The principles and mechanisms of protein folding were one of the major research topics and achievements of biochemical research in the last century. For decades, Anfinsen’s model, which explained protein structure by thermodynamic principles applying to the polypeptide’s inherent amino acid sequence (1), was to be found in the introductory sections of all textbooks in protein biochemistry. According to Anfinsen’s thermodynamic hypothesis, the structure with the lowest conformational Gibbs free energy was finally taken by each single polypeptide due to a thermodynamic and stereochemical selection for side chain relations that form most stable and effective enzymes or structural proteins (1). Beyond this individual selection for the energetically most optimized conformation, evolution also selected for amino acid sequences that energetically allowed the smoothest and most “frustration-free” folding processes via a thermodynamic “folding funnel” (1–3).

Whereas Anfinsen’s model preferred the side chain elements as preferential organizing structures, recent hypotheses have inversely proposed the backbone hydrogen bonds as the driving force behind protein folding (4). According to the former theory, the finally folded protein was assumed to attain a single defined structure and shape (1, 4), and the unfolded conditions were described as being represented by a structureless statistical coil with nearly indefinite conformations – a so-called “featureless energy landscape” (4). The latter model assumes that a protein selects during its folding process from a limited repertoire of stable scaffolds of backbone hydrogen bond-satisfied α-helices and β-strands (4). This also implies that unfolded proteins are not structureless, shoelace-like linear amino acid alignments as often depicted in cartoons for graphical reasons, but actually, at least in part, retain discrete and stable scaffolds.

Once the protein has attained its final conformation, the problem of stabilizing this structure arises. Hydrophobic interactions that press non-polar side chains into the center of the protein are assumed to be a major force in protein stabilization (5, 6). At the protein surface, polar interactions, mainly by hydrogen bonds of polar side chains and backbone structure, are assumed to be of similar importance (6). Salt bridges and covalent disulfide bonds were identified as further forces supporting the stability of proteins (6). Accordingly, all conditions that interfere with these stabilizing forces, including extreme temperature, salt concentrations, and redox conditions, may lead to protein misfolding.

Another aspect that must be taken into account when studying protein folding relates to the very different conditions found in viable cells when compared to test tube conditions. Considering the life-cycle of a protein, each protein begins as a growing polypeptide chain protruding from the ribosomal exit tunnel and with several of its future interacting amino acid binding partners not even yet attached to the growing chain of the nascent polymer. In these ribosomal exit tunnels, first molecular interactions and helical structures are formed, and evidence exists to support the notion that the speed of translation is regulated by slow translating codon sequences just to optimize these first folding processes (7). After leaving the ribosomal tunnel, nascent polypeptides are also directly welcomed by chaperoning protein complexes, which facilitate and further guide the folding process of newly synthesized proteins (8). It is believed that a high percentage of nascent proteins are subject to immediate degradation due to early folding errors (9). Since many nascent proteins are synthesized in parallel at polysomes, the temporal and spatial proximity of unfolded peptides brings the additional risk of protein aggregation (10). Moreover, as mentioned above, even incomplete folding intermediates and partially folded states may form energetically but not physiologically active metastable structures (11, 12). An immediate, perinatal guidance and chaperoning of newborn proteins is therefore essential to creating functional, integrative proteins and to avoiding misfolded, function-less polypeptides with potentially cytotoxic features.

Since protein structure and function are coupled, misfolded proteins are, at first, loss-of-function proteins that might reduce cell viability, in particular when generated in larger quantities. A more dangerous feature of misfolded proteins, however, lies in their strong tendency toward abnormal protein–protein interactions or aggregations, which is reflected by the involvement of misfolded proteins and their aggregates in several amyloidotic diseases, including neurodegenerative syndromes such as Alzheimer’s disease and Parkinson’s disease (13, 14). The fact that several of these intracellular and extracellular protein aggregates contain β-sheet-like structures and form filamentous structures also supports the notion that misfolded proteins are not necessarily structureless protein coils or unspecific aggregates, at least when they are formed by homogenous proteins as in the case of several neurodegenerative diseases (13). Paradoxically, these larger aggregates appear to reflect a cell protective mechanism so as to sequester or segregate smaller, but highly reactive, nucleation cores of condensing protein aggregates (13).

Unspecific hydrophobic interactions, in particular, have been held responsible for protein aggregations that form when terminally folded proteins lose their native conformation and expose buried hydrophobic side chains on their surface (15, 16). These hydrophobic interactions are also believed to be the most problematic issues with newly synthesized polypeptides on single ribosomes or polysomes (12). Once exposed to the surface, the hydrophobic structures will quickly find possible interaction partners. The intracellular milieu can be regarded as a “crowded environment” (17), fully packed with proteins in close contact and near to their solubility limit (8, 12). Thus, misfolded proteins not only aggregate among each other but may also attach to normal native proteins and inhibit their function and activity. Since such misfolding effects and interactions can also include nuclear DNA replication and repair enzymes (18), misfolded proteins may not only exert proteotoxic but also genotoxic effects, thereby endangering the entire cellular “interactome” (19) by interfering both with the integrity of the proteome (proteostasis) and the genome. Therefore, a misfolded protein is not simply a loss-of-function protein but also a promiscuous little villain that might act like a free radical, exerting uncontrolled danger to the cell.

The way in which cells deal with misfolded proteins strongly depends on the nature, strength, length, and location of the damage induced by the various insults. Management of misfolded proteins can be achieved by heat shock protein (HSP)-mediated protein renaturation (repair); proteasomal, lysosomal, or autophagosomal degradation (recycling); intracellular disposal (aggregation); or – in its last consequence if overwhelmed – by programed cell death (despair). In the following paragraphs, the cellular management of misfolded proteins is described and therapeutic options to induce misfolded proteins in cancer cells are presented.

## Hsp90 and Hsp90 Inhibitors

The best-known and evolutionarily most-conserved mechanism to protect against protein misfolding is the binding and refolding process mediated by so-called heat shock proteins (HSPs). HSPs recognize unfolded or misfolded proteins and facilitate their restructuring in either an ATP-dependent (large HSPs) or energy-independent manner (low weight HSPs). HSP of 90 kDa (hsp90) is a constitutively expressed HSP and is regarded as the most common and abundantly expressed HSP in eukaryotic cells (20, 21). Although commonly referred to as hsp90, it consists of a variety of isoforms that are encoding for cytosolic (hsp90α_1_, α_2_, β), mitochondrial (TRAP1), or endoplasmic reticulum (ER)-resident (GRP94) forms. Its primary function is less that of a stress response protein and more to bind to a certain group of client proteins unable to maintain a stable configuration without being assisted by hsp90 (20, 22, 23). Steroid hormone receptors (estrogen receptor, glucocorticoid receptor), cell cycle regulatory proteins (CDK4, cyclin D, polo-like kinase), and growth factor receptors and their downstream targets (epidermal growth factor receptor 1, HER2, AKT) are among the best-studied client proteins of hsp90 (20–22). Also, several cancer-specific mutations generating otherwise instable oncoproteins, such as mutant p53 or bcr-abl, rely on hsp90 chaperoning to keep them in a soluble form, thereby facilitating the extravagant but vulnerable “malignant lifestyle” of hsp90-addicted cancer cells (21, 24). Accordingly, hsp90 has been assumed to be a prominent target, in particular for hormone-responsive and growth factor receptor amplification-dependent cancer types.

The microbial antibiotics geldanamycin and radicicol are the prototypes of hsp90 inhibitors. Based on intolerable toxicity, these molecules had to be chemically modified for application in humans, and most of the ongoing clinical studies with hsp90 inhibitors are aimed at identifying semi-synthetic derivatives of these lead compounds with an acceptable risk profile. Unfortunately, most recent studies using geldanamycin derivatives have provided disappointing results because of toxicities and insufficient efficacy (22, 25–27). Studies with radicicol (resorcinol) derivatives, in particular with ganetespib, appear to be more promising because of fewer adverse effects (22, 25–27). Liver and ocular (retinal) toxicities have been described as main adverse effects of hsp90 inhibition, and appeared to be experienced less with ganetespib than with most of the first generation hsp90 inhibitors (28).

Since both geldanamycin and radicicol target the highly conserved and unique ATP-binding domain of hsp90, new synthetic inhibitors have also been generated by rational drug design (22, 25–27). However, none of the various natural or synthetic hsp90 inhibitors under investigation have yet provided convincing clinical data, and future studies will show whether hsp90 can eventually be added to the list of effective cancer targets.

## Hsp70, Hsp40, Hsp27, and HSF1

Hsp90 is assisted by several other HSPs and non-chaperoning co-factors, finally forming a large protein complex that recruits and releases client proteins in an energy-dependent manner (21, 22, 29). Client proteins for hsp90 are first bound to hsp70, which transfers the prospective client to hsp90 through the mediating help of an hsp70–hsp90 organizing protein (HOP). Binding of potential hsp90 client proteins to hsp70 is facilitated by its co-chaperone hsp40 (23, 30). Exposed hydrophobic amino acids, the typical feature of misfolded proteins, have been described as the main recognition signal for hsp70 proteins (15, 16, 31). Hsp70 proteins are not only supporter proteins for hsp90 but also represent a large chaperone family capable of acting independently of hsp90 and that can be found in all cellular compartments, including cytosol and nucleus (hsp70, hsp72, hsc70), mitochondria (GRP75 = mortalin), and the ER (GRP78 = BiP). Hsp70 chaperones may act on misfolded or nascent proteins either as “holders” or “folders” (31), which means that they prevent protein aggregation either by sheltering these aggregation-prone protein intermediates or by allowing these proteins to fold/refold into their native form in an assisted mechanism within a protected environment (31). Hsc70 (HSPA8) is a constitutively expressed major hsp70 isoform that is an essential factor for normal protein homeostasis even in unstressed cells (16). Misfolded proteins can also be destined by hsp70 proteins for their ultimate degradation. Proteins that expose KFERQ amino acid motifs on their surface during their unfolding process are preferentially bound by hsc70 and can be directed to lysosomes in a process called chaperone-mediated autophagy (CMA) (32, 33). In another mechanism of targeted protein degradation, interaction of hsc70 with the E3 ubiquitin ligase CHIP (carboxyl terminus of Hsc70-interacting protein) leads to ubiquitination of misfolded proteins and thus their destination of the ubiquitin-proteasome protein degradation pathway (34, 35). Since hsc70 is essential for normal protein homeostasis and its knock-out is lethal in mice (16, 36), hsc70 inhibition might not be an optimal target for cancer-specific induction of misfolded proteins. This contrasts with the inducible forms of hsp70 such as hsp72 (HSPA1), which are upregulated in a cell stress-specific manner and are often found to be constitutively overexpressed in cancer tissues (16, 36). Transcriptional activation of these inducible HSPs is mediated by the heat shock factor 1 (HSF1), which also regulates expression of hsp40 and the small HSP hsp27 by sharing a common promoter consensus sequence (heat shock response element) for HSF1 binding (37). HSF1 was also found to be constitutively activated in cancer tissues, modulating several cell cycle- and apoptosis-related pathways via its target genes (38–40). HSF1 itself is kept inactive in the cytosol by binding to hsp90, and the recruitment of hsp90 to misfolded proteins is considered a main activation mechanism to release monomeric HSF1 for its subsequent trimerization, post-translational activation, and nuclear translocation (24, 41). Also, since hsp90 inhibition causes hsp70 induction by HSF1 activation as a compensatory feed-back mechanism (24), combined inhibition of hsp90 and hsp70, or of hsp90 and HSF1 might be a more effective therapeutic approach for cancer treatment than single HSP targeting alone.

Indeed, several small-molecule inhibitors and aptamers for hsp70, hsp40, and hsp27 have been designed (16, 42–44), but most of them remain in pre-clinical development, or are either not applicable in humans or associated with intolerable side effects (16, 42–44). Notably, the natural bioflavonoid quercetin was shown to inhibit phosphorylation and transcriptional activity of the heat shock transcription factor HSF1, thus reducing HSP expression at its most basal level (45–48). This HSP and HSF1 inhibition may also contribute to the observed cancer-preventing effects of a flavonoid-rich diet, which includes fruits and vegetables. However, due to their low bioavailability, the concentrations of flavonoids needed to induce direct cytotoxic effects in cancer cells for (chemo-)therapeutic reasons are obviously not achievable in humans, even when applied as nutritional supplements (49). More effective and clinically more easily applicable inhibitors of HSF1 are therefore urgently sought. Promising HSF1 targeting strategies are currently under development, although are apparently not yet suited for clinical applications (24, 50, 51).

## Protein Ubiquitination and Proteasomal Degradation

Ubiquitin is a 76 amino acid polypeptide that can covalently be attached via its carboxy-terminus to free (lysyl) amino groups of proteins. Ubiquitination of proteins generates a cellular recognition motif that is involved in various functions ranging from transcription factor and protein kinase activation to DNA repair and protein degradation – depending on the extent and exact location of this post-translational modification (52, 53). Monoubiquitination of peptides of more than 20 amino acids was found to be a minimal requirement for protein degradation, but the canonical fourfold (poly-)ubiquitination with three further lysine (K48) side chain-linked ubiquitins appears to be most apt for an effective and rapid substrate recognition by the proteasome (54). This canonical polyubiquitin structure, as well as several other mixed polyubiquitin structures, can be recognized by the external 19S subunits of the 26S proteasome complex (54, 55). Prior to degradation of ubiquitinated proteins by the proteasomal 20S core subunit, the attached ubiquitin chains are released by the external 19S subunits for recycling, although they can also be co-degraded by the proteasome (56). After first passing the 19S subunit, the proteasomal target proteins are then unfolded in an energy-dependent manner and introduced into the narrow enzymatic cavity of proteasome for degradation. The barrel-shaped 20S proteasomal core complex contains three different proteolytic activities in duplicate (β1: caspase-like-, β2: tryptic-, and β5: chymotryptic activity), which initiate an efficient cleavage of the proteasomal target proteins into smaller peptides (57).

It is important to note that specific ubiquitination and ensuing proteasomal degradation is not an exclusive degradation mechanism of misfolded proteins but is also used to regulate the expression level of several native cell cycle regulatory proteins [cyclins, proliferating cell nuclear antigen (PCNA), p53], signaling pathway molecules (β-catenin, IκB), and survival factors (mcl-1) during the course of normal protein homeostasis and cell cycle progression (53, 55, 57, 58). Moreover, proteasomes are involved in protein maturation, including the processing and maturation of the NF-κB transcription factor subunit p50 and the drug-resistant protein MDR1 (57). Therefore, targeting proteasomal activity has not only been of interest for the generation of misfolded, cytotoxic proteins but also for interfering with the expression of proteins involved in several hallmarks of cancer, including cell cycle progression, signal transduction, and apoptosis.

## Proteasome Inhibitors

Bortezomib (PS-341, Velcade ™) has long been known as a paragon of a clinically applicable proteasome inhibitor. Bortezomib has been approved for the treatment of multiple myeloma and mantle cell lymphoma (55, 59, 60). The great expectations of transferring the success of bortezomib to non-hematological solid cancer types have unfortunately not yet been fulfilled. It has been suggested that the high antibody-producing capacity of myeloma cells and thus the need for an efficient proteasomal degradation system to cope with the recycling process of misfolded ER-generated antibodies [ER-associated degradation process (ERAD); see below] might contribute to the high sensitivity of myeloma cells to bortezomib (9, 60, 61). Originally, bortezomib was developed to inhibit the proteasomal degradation of the NF-κB inhibitor IκB, thus targeting the pro-inflammatory, but also cancer-promoting, effect of the NF-κB transcription factor (55, 60, 62). Recent insights indicate that the anti-tumoral effect of bortezomib is not only mediated by its NF-κB inhibitory activity but also by its ability to induce accumulation of misfolded proteins in the cytosol and the ER (60, 62–65). However, the use of bortezomib, even for highly sensitive multiple myeloma, is limited by its strong tendency to induce a proteasome inhibition-independent peripheral neuropathy by acting on neuronal mitochondria (61). Since neurodegenerative diseases are associated with protein misfolding and aggregation, the neuropathological effects of bortezomib might also be assumed to be mediated by the possible proteotoxic effects of bortezomib in neuronal cells. However, although proteasome inhibitor-induced neurodegeneration and inclusion body formation have been described in animal models, similarities between proteasome inhibitor-induced neurodegeneration and Parkinson’s disease-like histopathological features could not be established (66).

Due to the neurotoxic side effects of bortezomib and an increasing occurrence of refractory myelomas, much effort has been made to generate and identify new proteasome inhibitors with fewer adverse effects. In 2012, the irreversible proteasome inhibitor and epoxomicin derivate carfilzomib (Kyprolis™) was approved for treatment of bortezomib-resistant and relapsed multiple myeloma patients (62, 67). Although sharing the same target with bortezomib, the chymotryptic activity of the proteasome, carfilzomib was shown to overcome bortezomib resistance and to be associated with fewer neuropathological adverse effects than bortezomib (62, 67, 68) (Table 1). Since carfilzomib has the advantage of being an irreversible proteasome inhibitor, a more patient-friendly administration schedule with less frequent application and a better compliance may add to the advantages of carfilzomib.

**Table 1:** **Drugs described in this review and their mechanism of action (MOA), status of approval, and main adverse effects**.

Drug	MOA	Clinical application	Main adverse effects
Bortezomib (Velcade^TM^)	Proteasome inhibitor	Approval: multiple myeloma (MM); mantle cell lymphoma	Neutropenia and peripheral neuropathy (may lead to treatment abrogation)
		Trials: lung cancer; breast cancer; ovarian cancer; cervical cancer; prostate cancer; melanoma; colorectal cancer; pancreatic cancer; renal cancer; brain cancer; thyroid cancer; liver cancer; and diverse other solid cancers	
Carfilzomib (Kyprolis^TM^)	Proteasome inhibitor	Approval: refractory, bortezomib-resistant MM	Cardiotoxicity; pulmonary hypertension/lung problems; hepatic and renal toxicity; anemia. No pronounced neurotoxic effects.
		Trials: MM; lymphoma; unspecified solid cancers	
Nelfinavir (Viracept^TM^)	ER stress inducer; ROS generator	Approval: HIV infection	Gastrointestinal adverse effects; lipid metabolism disturbance; and insulin resistance (long-term)
		Trials: lung cancer; pancreatic cancer; cervical cancer; renal cancer; glioblastoma; colorectal cancer; MM	
Disulfiram (Antabuse^TM^)	Proteasome inhibitor; ROS generator	Approval: alcohol abuse	Alcohol intolerance; mild gastrointestinal, dermatologic, and ocular side effects. May affect nervous system.
		Trials: melanoma; breast cancer; liver cancer; glioblastoma; prostate cancer	
Ganetespib	HSP90 inhibitor	Trials: lung cancer; breast cancer; colorectal cancer; ovarian cancer; prostate cancer; liver cancer; melanoma; AML; MM	Neutropenia; nausea; fatigue; diarrhea
Ricolinostat (ACY-1215)	HDAC6 inhibitor	Trials: MM; lymphoma	Neutropenia
Chloroquine	Autophagy inhibitor	Approval: malaria treatment and prophylaxis; arthritis	Gastrointestinal and visual problems; renal and cardiac toxicity; pruritus
		Trials: lung cancer; breast cancer; glioblastoma; MM	

*MM, multiple myeloma; AML, acute myeloid leukemia; ROS, reactive oxygen species*.

Additional newly generated proteasome inhibitors have been developed, which are currently being tested in clinical trials (53, 61, 65, 69) and will hopefully soon expand our limited arsenal of clinically applicable proteasome inhibitors. Unfortunately, both bortezomib and carfilzomib are extremely expensive medications and result in high therapy costs (68), so more cost-effective proteasome inhibitors are urgently needed. Interestingly, several natural polyphenols have been described as exerting proteasome-inhibitory activity (70, 71). As mentioned, however, the clinical use of flavonoids in cancer treatment is limited due to their poor bioavailability (49, 72), although the compounds may be used as lead molecules for the development of proteasome inhibitors. Notably, natural flavonoids have also been identified as reducing the therapeutic efficacy of bortezomib by a direct chemical interaction of vicinal hydroxyl groups of flavonoids with the boronic acid residue of bortezomib, thus leading to disregard of a combination therapy of bortezomib with flavonoids (73).

Instead, other combination therapies of bortezomib, in particular with hsp90 inhibitors appear to be more appropriate for therapeutic applications. A clinical study with the geldanamycin-derivate 17-AAG and bortezomib, however, revealed no clinical response but severe adverse effects resulted from this combination in acute myeloid leukemia patients (74). By contrast, a study with bortezomib and the hsp90 inhibitor tanespimycin in patients with relapsed and refractory multiple myeloma (75) revealed an objective response rate of 27% and was described to be well tolerated (75). In patients with diverse solid cancer receiving similar doses of this drug combination, however, no objective response was observed (76).

## Disulfiram

Another prospective tool for cost-effective proteasome inhibition is the repurposed use of approved and (if ever possible) patent-free drugs that have also proved to cause proteasome inhibition as an off-target effect. The alcohol-deterring drug disulfiram (Antabuse) has been shown to be a highly active proteasome inhibitor (77, 78), in particular when combined with endogenous or exogenous copper ions (79). Treatment of cancer cells with disulfiram caused all characteristics of efficient proteasome inhibition, including NF-κB inhibition and accumulation of polyubiquitinated proteins and protein aggregates (77, 78, 80). Notably, non-malignant cells responded less to disulfiram treatment, in accordance with the low toxicity profile of this well-tolerated drug, as also experienced in course of its use for anti-alcoholism for over 60 years (79). In addition to its proteasome-inhibitory effect, disulfiram/copper has been shown to generate reactive oxygen species in cancer cells, further contributing to the large amount of misfolded and aggregated proteins seen after disulfiram treatment (78, 80). Disulfiram is degraded *in vivo* into various reactive metabolites, one of which, diethyldithiocarbonate, is able to covalently bind to reactive thiol groups of proteins and to inactivate cancer-promoting kinases and drug-resistance-conferring enzymes such as protein kinase C (PK-C), P-glycoprotein (MDR1), DNA methyltransferases (DNMT), and aldehyde dehydrogenase (ALDH) (81). Inhibition of ALDH has long been held responsible for the alcohol-deterring effect of disulfiram, but ALDH is also known to belong to a group of cancer-related genes referred to as cancer stem cell genes (81). Since cancer stem cell genes have been held responsible for drug resistance and cancer recurrence, targeting of ALDH1 by disulfiram may also tackle the highly drug-resistant cancer stem cell subpopulation. These promising pleiotropic, but mostly cancer cell specific, proteotoxic effects of disulfiram recently instigated several clinical trials with disulfiram in cancer patients (79, 82).

## Aggresome Formation and Re-Solubilization: Role of HDAC6

**Figure 1:**
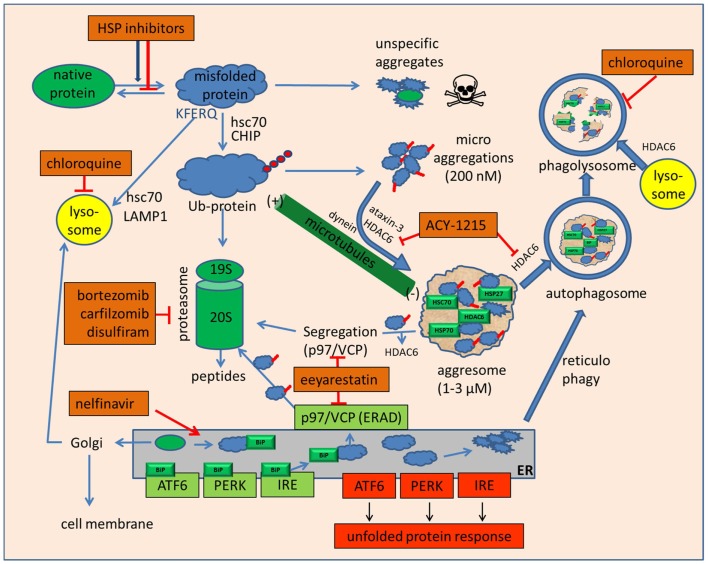
**Drugs that inhibit folding or disposal of misfolded proteins**. Native mature proteins, nascent proteins, or misfolded proteins can be prevented from folding or refolding by small and large heat shock protein inhibitors, of which the hsp90 inhibitors based on geldanamycin and radicicol are currently the most advanced in clinical studies. To avoid accumulation of misfolded proteins, their degradation can be mediated by hsc70, which may divert these proteins either to lysosomes to be degraded by chaperone-mediated autophagy or, by specific ubiquitination, to proteasomes. In case of unmanageable amounts of misfolded proteins or proteasome inhibition, unspecific aggregation of these proteins may occur. These highly cytotoxic small protein aggregates can be sequestered in an HDAC6-dependent manner with the help of microtubules into large, perinuclear aggresomes near the microtubule organizing center. Inhibition of HDAC6 by tubacin, tubastatin, or ACY-1215 inhibits disposal of microaggregates and may enhance the toxicity of bortezomib and HSP inhibitors. Aggresomes are not final deposits but may be re-dissolved either *en bloc* by macroautophagy or by molecular segregation via p97/VCP and final degradation by proteasomes. The p97/VCP inhibitor eeyarestatin may inhibit this degradation pathway but also aggresome formation by interfering with ataxin-3. Eeyarestatin also inhibits both anterograde and retrograde transport of ER proteins. Induction of ER stress induces the unfolded protein response by sequestering BiP from membrane receptors ATF6, PERK, and IRE. The unfolded protein response leads to cytoprotective chaperone synthesis but also to the expression of pro-apoptotic CHOP, NOXA, and c-JUN-kinase activation in case of prolonged or unmanageable ER stress. ER stress may also be alleviated by autophagy, whose efficacy can be inhibited by the lysosomotropic anti-malaria drug chloroquine.

As depicted above, proteasome and HSP inhibition will eventually lead to the accumulation of misfolded and polyubiquitinated proteins. Based on their inherent cohesive properties mediated by their exposed hydrophobic surfaces, both ubiquitinated and non-ubiquitinated misfolded proteins tend to adhere as small aggregates (Figure 1). Individual ubiquitinated proteins and small ubiquitinated aggregates can be recognized by specific ubiquitin-binding proteins such as HDAC6 via its zinc finger ubiquitin-binding domain. HDAC6 is an unusual histone deacetylase located in the cytosol that regulates microtubule acetylation and is also able to bind ubiquitinated proteins. Based on HDAC6’s additional ability to bind to microtubule motor protein dynein, these aggregates are actively transported along the microtubular system into perinuclear aggregates around the microtubule organizing center (MTOC) (10, 83, 84). Recognition of small, scattered ubiquitinated aggregates by HDAC6 has been described as being mediated by unanchored ubiquitin chains, which are generated by aggregate-attached ubiquitin ligase ataxin-3 (85). Whereas proteasomal target proteins are primarily tagged by K-48 (lysine-48) linked ubiquitins; K-63 linked ubiquitin chains appear to be a preferential modification for aggresomal targeting by HDAC6 and were assumed to mediate a redirection from proteasomal degradation to aggresome formation in the case of proteasomal inhibition or overload (86). Accordingly, aggresome formation is not an unspecific protein aggregation but a specific, ubiquitin-controlled sorting process. Furthermore, these aggresomes consist not only of misfolded and deposited proteins but have also been shown to contain a large amount of associated HSPs and ubiquitin-binding proteins, including HDAC6 [Figure 1; (10, 83, 84)]. Aggresomes contain, and are also surrounded by, large numbers of proteasomes (10, 83, 84), which help to resolubilize these aggregates not only through their intrinsic proteasomal digestion but also by generating unanchored K63-branched polyubiquitin chains, which then stimulate HDAC6-mediated autophagy, another cellular disposal mechanism in involving HDAC6 (87). Notably, HDAC6 has also been shown to control further maturation of autophagic vesicles by stimulating autophagosome–lysosome fusion (Figure 1) in a manner different from the normal autophagosome–lysosome fusion process (88).

The HDAC6 multitalent also exerts its deacetylase activity on hsp90 and modifies hsp90 client binding by facilitating its chaperoning of steroid hormone receptors and HSF1 (89–91). Recruitment of HDAC6 to ubiquitinated proteins leads to the dissociation of the repressive HDAC6/hsp90/HSF1 complex (91) and allows the release of transcriptionally active HSF1 to the nucleus. The engagement of HDAC6 at the aggresome–autophagy pathway hence also indirectly facilitates HSF1 activity. p97/VCP (valosin-containing protein), another binding partner of HDAC6 and itself a multi-interactive, ATP-dependent chaperone (92–94), is assumed to be involved not only in the specific separation of hsp90 and HSF1 by its “segregase” activity but also in the binding and remodeling of polyubiquitinated proteins before their delivery to the proteasome (93–95). Additionally, p97/VCP dissociates polyubiquitinated proteins bound to HDAC6 (91). Accumulation of polyubiquitinated proteins thus leads to HDAC6-dependent HSF1 activation and HSP induction, p97/VCP-dependent recruitment and “preparation” of polyubiquitinated proteins to proteasomes, and, in the case of pharmacological proteasome inhibition or physiological overload, to an HDAC6-dependent detoxification of polyubiquitinated proteins by the aggresome/autophagy pathway.

## Pharmacological Inhibition of Aggresome Formation: HDAC6 Inhibitors

The central involvement of HDAC6 in aggresome formation and clearance makes HDAC6 one of the most interesting druggable targets for the induction of proteotoxicity in cancer cells. Also, HDAC6 has been found to be overexpressed in various cancer tissues, associated with advanced cancer stages and increased neoplastic transformation (96). Several pan-histone deacetylase inhibitors have been developed and tested in clinical studies for a variety of diseases, including different types of cancer (97, 98). Although hematological malignancies responded best to most of the already clinically tested pan-histone deacetylase inhibitors, the efficacy on solid cancer types was disappointingly poor and also associated with intolerable side effects (98). The unforeseeable pleiotropic epigenetic mechanism caused by non-specific (nuclear) histone deacetylase inhibitors may also limit their application for use in cancer treatment or HDAC6 inhibition, and has led to the search for selective HDAC6 inhibitors with no inhibitory effects on transcription modifying histone deacetylases. Through screening of small molecules under the rationale of selecting for tubulin deacetylase inhibitors with no cross-reactive histone deacetylase activity, the HDAC6 inhibitor tubacin was identified, and suggested for use in the treatment of neurodegenerative diseases or to reduce cancer cell migration and angiogenesis (99). Hideshima et al. then proved the hypothesis that the combined use of bortezomib with tubacin leads to an accumulation of non-disposed cytotoxic proteins and aggregates in cancer cells (100). Indeed, a synergistic effect of these two drugs against multiple myeloma cells could be observed with no detectable toxic effect on peripheral blood mononuclear cells (100). This and follow-up studies also revealed the efficacy of tubacin as a single agent against leukemia cells (100, 101) and a chemo-sensitizing effect on cytotoxic drugs in breast- and prostate-cancer cells (102).

Another specific HDAC6 inhibitor, tubastatin A (103) revealed promising effects in neurodegenerative diseases (104) but displayed apparently limited efficacy on solid cancer cells (105). A very interesting new and orally applicable HDAC6 inhibitor, ACY-1215 (ricolinostat), has recently been developed, which causes ER stress and caspase-dependent apoptosis in synergy with bortezomib in multiple myeloma cells (106). The promising pre-clinical data and the oral applicability have instigated several clinical studies to test the efficacy of ACY-1215 either as a single agent or in combination with bortezomib in patients with multiple myeloma and other lymphoid malignancies^1^.

## Endoplasmic Reticulum Stress

Besides the cytosol, the ER is a major site for protein synthesis, in particular for those proteins destined for extracellular secretion, the cell membrane, or their retention within the endomembrane system. At the rough ER, nascent proteins are co-translationally transported across the ER membrane into the ER lumen (107), where they immediately encounter ER-resident chaperones, most prominently represented by hsp70 family member BiP/GRP78 and hsp90 family member GRP94 to help proper protein folding (15, 108). Most of these proteins also undergo post-translational modifications, including N- or O-linked glycosylation or protein disulfide bridge-building (109, 110), thereby adding further mechanisms of protein stabilization but also challenges for proper protein folding.

Similar to the situation in cytosolic protein biosynthesis, a large proportion of nascent proteins in the ER are assumed to misfold and to go “off-pathway” even under normal physiological conditions. Furthermore, the ER lumen, narrowly sandwiched between two phospholipid membranes, has been described as an even more densely crowded environment than the cytosol, additionally facilitating unspecific protein attachments and aggregations (15). Since, with the exception of bulk reticulophagy, the lumen of the ER contains no endogenous protein degradation system, and the anterograde transport of ER proteins to the Golgi, lysosomes, endosomes, or the extracellular environment requires properly folded proteins, a retrograde transport of ER proteins into the cytosol remains the only possible mechanism of preventing misfolded protein accumulation within the ER. In this ERAD, misfolded proteins are re-exported across the ER membrane by a specific multi protein complex, ubiquitinated by ER membrane-integrated ubiquitin ligases, and finally become degraded by cytosolic proteasomes (111, 112). Notably, association of the cytosolic p97/VCP protein, an important interacting partner with HDAC6, has also been described as being an essential factor for driving the luminal proteins through the ER membrane pore complex into the cytosol (92, 112).

Accordingly, all agents and conditions that interfere with these folding, maturation, and retranslocation processes can lead to protein misfolding and aggregation within this sensitive organelle. Chemicals that act as glycosylation inhibitors (tunicamycin), calcium ionophore inhibitors (A23187, thapsigargin), heavy metal ions (cadmium, lead), reducing agents (dithiothreitol), as well as conditions like hypoxia or oxidative stress, all lead to a phenomenon called ER stress (113–116). In the ER-stress response, a triad of ER membrane-resident signaling receptors and transducers, IRE1, ATF6, and PERK1, become activated and lead to the transcriptional activation of cytosolic and ER-resident chaperones to cope with the increasing number of misfolded proteins. Induction of autophagy (reticulophagy; ER-phagy) may also occur and supports the removal of damaged regions of the ER (117). Under very intensive or even unmanageable ER-stress conditions, a variety of pro-apoptotic pathways ensue, including CHOP induction, c-JUN-kinase activation, and caspase cleavage (118–120), which eventually prevails over the cytoprotective arm of the ER-stress response and may lead to apoptosis. Targeting of protein folding within the ER is therefore a very promising strategy to induce apoptosis in cancer cells, in particular in those cancer cells characterized by an unphysiologically high protein secretion rate, such as, for example, multiple myeloma cells. Whereas the above-mentioned drugs such as tunicamycin or thapsigargin are valuable tools for cell biology studies, they display unacceptable toxicities in humans and are not suited for therapeutic applications. Interestingly, several already established drugs used for non-cancerous diseases have been described as inducing ER stress at pharmacologically relevant concentrations in humans as an off-target effect (113, 116). The non-steroidal anti-inflammatory COX-2 inhibitor celecoxib is an approved drug to treat various forms of arthritis and pain, but has also been described as exerting ER stress by functioning as a SERCA (sarco/ER Ca^2+^ ATPase) inhibitor (113, 116). However, although well tolerated in humans, the ER-stress-inducing ability of celecoxib seems to be weaker than that of direct SERCA inhibitors such as thapsigargin, and the usefulness of celecoxib against advanced cancer has been questioned (116). Various HIV protease inhibitors have been described as inducing ER stress in human tissue cells as a side effect (121–123). In particular the HIV drugs lopinavir, saquinavir, and nelfinavir appear to be potent inducers of the ER-stress reaction, leading to a focused interest in these drugs for the induction of ER stress and apoptosis in cancer cells (116, 124–128). In fact, with currently over 27 clinical studies in cancer patients^2^, nelfinavir, either used as a single agent or in combination therapy, is on the list of the most promising prospective candidates to induce selective proteotoxicity in cancer cells at pharmacologically relevant concentrations. Although the exact mechanism by which nelfinavir induces ER stress is not yet clear, it was shown that nelfinavir causes the upregulation of cytosolic and ER-resident HSPs, and induces apoptosis in cancer cells associated with caspase activation and induction of the pro-apoptotic transcription factor CHOP (125, 126). Nelfinavir was also shown to be combinable with bortezomib to enhance its activity on cancer cells (129). Since the retrograde transport of misfolded ER proteins is inhibited by the p97/VCP inhibitor eeyarestatin (130, 131), we recently tested the combination of eeyarestatin with nelfinavir but found no synergistic effect between these two agents in cervical cancer cells (132). In contrast, eeyarestatin markedly sensitized cervical cancer cells to bortezomib treatment (132), which was also observed in preceding studies in which eeyarestatin was used to augment the ER-stress-inducing ability of bortezomib in leukemia cells (131). The combination of bortezomib with eeyarestatin massively increased the accumulation of polyubiquitinated proteins in cancer cells, induced macroautophagy, and activated the pro-apoptotic ATF4/CHOP/NOXA-mediated pathway of the ER-stress response (131, 132). These cytotoxic effects were also observed when eeyarestatin was applied as a single agent to cancer cells (131, 132), but not in normal blood cells (131). Eeyarestatin as a single agent might therefore be of interest as an alternative to bortezomib treatment, and has also successfully been applied in a xenograft model (133), although little is known of its pharmacological effects in humans. In combination with bortezomib, eeyarestatin might help to reduce the amount of bortezomib needed to induce cytotoxic effects in solid cancer cells (132).

## Therapeutic Hyperthermia

Heat application is the most direct method to generate misfolded proteins in cancer cells. Indeed, local, regional, and whole body hyperthermia is a practiced cancer treatment modality, although in most cases is used to support and improve the efficacy of radiotherapy and chemotherapy (134, 135). Thanks to the basic or inducible expression of HSPs, normal body cells, and cancer cells can withstand temperatures well above the core body temperature of 37°C. In the oncologically relevant therapeutic temperature range of 40–47°C, a time and temperature relation exists that defines whether cells will survive or undergo cell death after heat exposure (136, 137). Temperatures between 40.5 and 42°C were described to cause only weak heat stress and no extensive apoptosis in cancer cells, with the extent of apoptosis rapidly increasing when temperatures were elevated from 42 to 45°C (137, 138). Further temperature elevation to 46–47°C then mainly causes unspecific, heat-induced necrosis alone (138). Interestingly, at these elevated temperatures, the quantity of HSPs is not further increased but instead decreased (135). Instead, some HSPs, in particular hsp70, are translocated to the cell membrane or are released by necrotic cells into the extracellular milieu and are able to elicit an immunologic response against surviving cancer cells (135, 139). The cellular response to heat is also highly variable among cell types. Temperatures of more than 48–50°C are believed to be generally intolerable to cancer as well as healthy body cells and can be used for local thermal ablation (137) in cases where surgery is either not possible or not wanted by the patient.

Although the whole cell and all its organelles are subjected to the detrimental heat impact, the nucleus seems to be one of the organelles most affected by hyperthermia. Non-histone nuclear proteins appear to be particularly sensitive to heat inactivation, resulting in denaturation of these nuclear proteins and their aggregation among each other, but also to correctly folded, aggregation-sensitive native proteins within the nucleus (18). Overall, this may lead to nuclear enzyme inactivation, DNA masking by aggregated proteins, impairment of DNA synthesis and repair, and a mitotic catastrophe in case cells continue to divide (18, 136, 137). In addition to an additive pro-apoptotic effect of heat to the cytotoxic insults of radiation or chemotherapeutic drugs on cancer cells (136), the supportive effect of hyperthermia is probably also due to the inhibitory effect of heat on DNA repair and drug detoxifying enzymes. However, despite several precious and specific anti-cancer effects, hyperthermia, as mentioned above, has not been established as a single cancer treatment but is primarily used as a supportive modality in conjunction with chemo- and radiotherapy (134, 135).

## Conclusion and Perspectives

Induction of proteotoxicity through the accumulation of misfolded proteins has evolved as a new treatment modality in the fight against cancer. Clinically approved drugs such as bortezomib and carfilzomib provide evidence of the functionality of this approach. Newly developed agents like the HDAC6 inhibitor ACY-1215 or repurposed drugs like nelfinavir or disulfiram are currently being tested in clinical trials with cancer patients and will hopefully further broaden our arsenal of anti-cancer drugs. Notably, most proteotoxic agents that have been approved or are in clinical trials target the ubiquitin-proteasome-system (UPS) and are mainly effective in multiple myeloma cells, which rely on a functional ER/ERAD/UPS for excessive and proper antibody production. Similarly, it can be assumed that other cancer cell types with a marked secretory phenotype may also be affected by ER/ERAD/UPS inhibitors. In accordance with this notion, a recent dose-escalating Phase Ia study with nelfinavir as a single agent, that covered a large variety of solid cancer entities, revealed response rates primarily in patients with neuroendocrine tumors (140). In most other solid cancer types, however, the chemo-sensitizing or combination effects of proteotoxic drugs may prevail, and have become the focus of an increasing number of very promising clinical and pre-clinical studies.

## Conflict of Interest Statement

The authors declare that the research was conducted in the absence of any commercial or financial relationships that could be construed as a potential conflict of interest.

## Footnotes

^1^http://www.clinicaltrials.com

^2^www.clinicaltrials.gov
